# Select nutrients, progesterone, and interferon tau affect conceptus metabolism and development

**DOI:** 10.1111/j.1749-6632.2012.06741.x

**Published:** 2012-10-10

**Authors:** Fuller W Bazer, Jingyoung Kim, Gwonhwa Song, Hakhyun Ka, Carmen D Tekwe, Guoyao Wu

**Affiliations:** 1 Department of Animal Science, Texas A&M UniversityCollege Station, Texas; 2 Department of Agricultural Biotechnology, Seoul National UniversitySeoul, Republic of Korea; 3 Department of Biological Resources and Technology, Yonsei UniversityWonju, Republic of Korea; 4 Faculty of Nutrition, Texas A&M UniversityCollege Station, Texas

**Keywords:** amino acids, fat, glucose, interferon tau, pregnancy, uterus

## Abstract

Interferon tau (IFNT), a novel multifunctional type I interferon secreted by trophectoderm, is the pregnancy recognition signal in ruminants that also has antiviral, antiproliferative, and immunomodulatory bioactivities. IFNT, with progesterone, affects availability of the metabolic substrate in the uterine lumen by inducing expression of genes for transport of select nutrients into the uterine lumen that activate mammalian target of rapamycin (mTOR) cell signaling responsible for proliferation, migration, and protein synthesis by conceptus trophectoderm. As an immunomodulatory protein, IFNT induces an anti-inflammatory state affecting metabolic events that decrease adiposity and glutamine:fructose-6-phosphate amidotransferase 1 activity, while increasing insulin sensitivity, nitric oxide production by endothelial cells, and brown adipose tissue in rats. This short review focuses on effects of IFNT and progesterone affecting transport of select nutrients into the uterine lumen to stimulate mTOR cell signaling required for conceptus development, as well as effects of IFNT on the immune system and adiposity in rats with respect to its potential therapeutic value in reducing obesity.

## Interferon tau, immunological tolerance, and metabolic diseases

Interferon tau (IFNT), a novel type 1 interferon secreted by mononuclear trophectoderm cells of ruminant conceptuses (embryo and its extraembryonic membranes), is the pregnancy recognition signal.^1^^,^%^2^ In uterine epithelial cells IFNT silences transcription of genes for receptors for estrogens and oxytocin to abrogate oxytocin-induced pulsatile secretion of luteolytic prostaglandin F_2α_ (PGF) to ensure maintenance of functional corpora lutea for secretion of progesterone (P4), the hormone of pregnancy. IFNT also has antiproliferative, antiviral, and immunomodulatory effects, but without the cytotoxic effects associated with other type I interferons (e.g., interferon alpha).^1^^,^%^2^

IFNT induces immunological tolerance in several animal models; therefore, there is interest in its therapeutic value for treatment of inflammatory diseases. For example, matings between DBA/2 and C57BL/6 mice result in high rates of fetal resorption, but treatment of dams with exogenous IFNT prevents this pregnancy wastage in an interleukin 10 (IL-10)–dependent manner.^3^^,^^4^ In the experimental allergic encephalomyelitis (EAE) mouse model, IFNT suppresses EAE by stimulating IL-10—a T helper cell (Th2) response.^5^^,^^6^ Treatment of NOD mice with IFNT orally, intraperitoneally, or subcutaneously delays or inhibits development of diabetes by reducing inflammation of beta cells in pancreatic islets and inducing anti-inflammatory mechanisms.^7^ In pregnant sheep, IFNT silences expression of MHC class I and β_2_ microglobulin genes in uterine luminal (LE) and superficial glandular epithelia (sGE) in order to prevent maternal immune responses to the conceptus.^8^

We produced a synthetic gene for IFNT and use a high-yield *Pichia pastoris* expression system to produce recombinant ovine IFNT (roIFNT) for our studies.^9^ Results from human clinical trials indicate that daily oral doses of 3 mg roIFNT (three times each day) for up to nine months is safe and well tolerated by patients.^10^ Available results indicate that IFNT treatment leads to an anti-inflammatory phenotype of tolerance that may mitigate against progression of obesity, type 2 diabetes (T2D), and other inflammatory diseases.

## Obesity, an inflammatory disease, and interferon tau

Obesity results from a chronic imbalance between energy intake and expenditure, but it is also an autoimmune disease responsible for a major global health crisis as a risk factor for insulin resistance, T2D, atherosclerosis, stroke, hypertension, and cancer.^11^ Obesity in white adipose tissue (WAT) involves chronic local inflammation linked to the innate immune system, particularly macrophages and lymphocytes, which predispose individuals to metabolic syndrome.^12^^,^^13^ The central hypothesis of our research on obesity is that IFNT suppresses production of proinflammatory cytokines and adipokines in WAT, which are central to development of an inflammatory state that is central to development of obesity, T2D, and metabolic syndrome.

## Effects of IFNT on obesity and onset of diabetes

Research in progress in our laboratories involves Zucker diabetic fatty (ZDF) rat and Sprague–Dawley rats administered IFNT orally. In general, IFNT reduces concentrations of branched-chain amino acids, decreases WAT, and increases brown adipose tissue. Extending research with ZDF rats, we reported that arginine enhances expression of IFNT in the ovine conceptus^14^ and decreases maternal white fat in obese sheep.^15^

## Nutrition and successful outcomes of pregnancy

Insufficient delivery of nutrients to the developing conceptus results in intrauterine growth restriction (IUGR), a significant social and economic problem of global importance. In addition to increased risk of perinatal morbidity and mortality associated with nutrient restriction, IUGR puts offspring at increased risk for metabolic diseases later in life, referred to as developmental origins of adult disease.^16^ Therefore, a primary goal of our research is to understand mechanisms whereby select nutrients transported into the uterine lumen serve to optimize survival, growth, and development of the mammalian conceptus.

## Conceptus development and pregnancy recognition in sheep

Sheep embryos enter the uterus on day 3, develop to spherical blastocysts, and, after hatching from the zona pellucida, transform from spherical to tubular and filamentous conceptuses (embryo and associated extraembryonic membranes) between days 12 and 15 of pregnancy, with extraembryonic membranes extending into the contralateral uterine horn between days 16 and 20 of pregnancy.^17^ Elongation of ovine conceptuses is a prerequisite for implantation involving apposition and adhesion between trophectoderm and uterine LE/sGE and for placentation and a successful outcome of pregnancy. A transient loss of uterine LE allows intimate contact between conceptus trophectoderm and uterine stromal cells until about day 25 when uterine LE is restored in the intercaruncular endometrium.^18^ All mammalian uteri contain uterine glands that produce or selectively transport a complex array of proteins and other molecules known collectively as histotroph. Histotroph is required for elongation and development of conceptuses, and ewes lacking uterine glands and histotroph fail to exhibit normal estrous cycles or maintain pregnancy beyond day 14.^19^

Ovine IFNT plays a central role in molecular mechanisms that underlie both pregnancy recognition signaling and establishment and maintenance of a uterine environment conducive to successful outcomes of pregnancy.^20^ IFNT silences transcription of estrogen receptor alpha (*ESR1*) and, therefore, ESR1-dependent expression of the oxytocin receptor (*OXTR*) gene in uterine LE/sGE, which abrogates development of the endometrial luteolytic mechanism that requires oxytocin-induced release of luteolytic pulses of prostaglandin F_2α_ (PGF) by uterine LE/sGE.^21^ Importantly, IFNT also acts in concert with P4 to induce expression of genes for transport and/or secretion of histotroph that includes nutrients, such as glucose and arginine (Arg), that activate the mTOR nutrient-sensing cell signaling pathway to stimulate proliferation, migration, and differentiation of trophectoderm cells, as well as increased translation of mRNAs essential for growth and development of the conceptus.

The amounts of Arg, leucine (Leu), glutamine (Gln), and glucose in uterine histotroph increase significantly between days 10 and 15 of pregnancy due to effects of P4 and IFNT to increase expression of genes for their respective transporters specifically in uterine LE/sGE. In day-16 ovine conceptus explant cultures Arg increases GTP cyclohydrolase 1 (*GCH1*) mRNA; both Arg and glucose increase ornithine decarboxylase (*ODC1*), nitric oxide synthase 2 (*NOS2*), and GCH1 proteins; and Arg increases IFNT. GCH1 is the rate-limiting enzyme for synthesis of tetrahydrobiopterin, an essential cofactor for all NOS isoforms. Arg can be metabolized to nitric oxide (NO) by NOS, and to polyamines by ODC1 via arginase, both of which stimulate proliferation of ovine trophectoderm (oTr) cells.^22^ Secreted phosphoprotein 1 (SPP1), secreted by uterine GE in response to P4, is present in ovine uterine histotroph and increases migration, focal adhesion assembly, adhesion, and, perhaps, T regulatory (T_reg_) cell proliferation.

### Select nutrients and mTOR cell signaling in the pregnant uterus

The mTOR cell-signaling pathway plays an important role in regulation of cell growth and metabolism in response to growth factors and nutrients. MTOR is an evolutionarily conserved serine/threonine kinase located downstream of PI3K and AKT1, which controls cell growth and proliferation through activation of ribosomal protein S6 kinase (RPS6K) to phosphorylate RPS6 and, ultimately, regulate protein synthesis,^23^^,^^24^ as well as initiate mRNA translation, ribosome synthesis, expression of metabolism-related genes, autophagy, and cytoskeletal reorganization.^25^ The mTOR pathway is a “nutrient sensing system” responsive to molecules that include SPP1, insulin-like growth factor 2 (IGF2), glucose, and selected amino acids that are required for blastocyst/conceptus development.^26^^–^^30^ Homozygous *Frap1*-null mice die shortly after implantation due to impaired cell proliferation and hypertrophy in both the embryonic disc and trophoblast.^31^

The mTOR cell-signaling pathway is a prominent component of the peri-implantation intrauterine environment in sheep.^32^^–^^42^ Progesterone and IFNT stimulate expression of *RHEB* and *EIF4EBP1* in ovine uterine endometria, resulting in increased abundance of mRNAs for *RICTOR*, *RHEB,* and *EIF4EBP1*, as well as the RHEB protein, which is coordinate with rapid growth and development of ovine conceptuses. The abundance of *LST8*, *MAPKAP1*, *RHEB,* and *EIF4EBP1* mRNAs in ovine conceptuses during the peri-implantation period of pregnancy increases coincident with their growth and development, and mTORC1 is abundant in the cytoplasm and phosphorylated mTOR is abundant in nuclei of trophectoderm and endoderm cells to stimulate proliferation and migration of these cells, as well as protein synthesis. Our research with pregnant and cyclic ewes is focused on nutrients in uterine histotroph that affect mTOR cell signaling, as well as expression of transporters for glucose and amino acids and related enzymes.

Results from studies of pregnant and cyclic ewes indicate that total recoverable glucose, Arg, Leu, and Gln, as well as glutathione, calcium, and sodium increase in uterine fluids of pregnant, but not in uterine fluids of cyclic ewes, between days 10 and 16 after onset of estrus.^32^^–^^38^ This is due to tissue and cell-specific expression of select facilitative (*SLC2A1*, *SLC2A3,* and *SLC2A4)* and sodium-dependent glucose transporters (*SLC5A1* and *SLC5A11*), cationic amino acid transporters (*SLC7A1, SLC7A2, SLC7A3,* and *SLC7A6*), neutral amino acid transporters (*SLC1A4, SLC1A5, SLC3A1, SLC6A14*, *SLC6A19*, *SLC7A8, SLC38A3, SLC38A6, SLC7A8,* and *SLC43A2*), and acidic amino acid transporters (*SLC1A1*, *SLC1A2,* and *SLC1A3*) that transport the various amino acids and glucose into the uterine lumen. Among these genes *SLC2A3* and *SLC7A6* are detectable only in trophectoderm and endoderm of conceptuses specifically for transport of glucose and cationic amino acids such as arginine from the uterine lumen and into those cells. The abundance of mRNAs for *SLC2A1*, *SLC2A4, SLC5A1, SLC5A11, SLC7A1, SLC7A2, SLC1A4, SLC1A5, SLC43A2,* and *SLC1A3* in ovine uterine endometria varies due to day of the estrous cycle and pregnancy. Expression of mRNAs for *SLC1A5*, *SLC2A1, SLC5A11,* and *SLC7A1* in endometria is induced by P4 and further stimulated by IFNT. Collectively, these results indicate the presence of the mRNAs and proteins associated with both mTORC1 and mTORC2 cell signaling in the ovine uterus and conceptus, as well as nutrient transporters for delivery of Arg, Leu, Gln, and glucose into the uterine lumen, and then into the various cell types of the conceptuses.

Research on the effects of the estrous cycle, pregnancy, P4, and IFNT on expression of *NOS1*, *NOS2A*, *NOS3*, *GCH1,* and *ODC1* revealed that both *NOS1* and *ODC1* are expressed by uterine LE/sGE, while NOS3 is most abundant in conceptus trophectoderm and endoderm.^36^ Expression of *GCH1* for synthesis of tetrahydrobiopterin, the cofactor for all *NOS* isoforms for NO production, as well as *ODC1* and *NOS1* is more abundant in conceptuses than cells of the uterine endometrium. P4 stimulates expression of *NOS1* and *GCH1*, while IFNT inhibits expression of *NOS1*. Therefore, key molecules for metabolism of Arg (NOS2, NOS3) and ornithine (ODC1) are present to account for production of NO and polyamines that are critical to conceptus growth and development.

## Pathways for Arg-mediated effects on proliferation and migration of ovine trophectoderm cells

Arg is most stimulatory to proliferation, migration, and protein synthesis in our established oTr cell line.^14^^,^^39^^–^^41^ Arg mediates its effects in oTr cells by increasing (1) phosphorylation of RPS6K in a dose-dependent manner; (2) phosphorylated forms of AKT1, RPS6K, and RPS6 over basal levels; (3) nuclear phosphorylated RPS6K and cytoplasmic phosphorylated RPS6; and (4) proliferation and migration of oTr cells. Phosphorylation of RPS6K and RPS6 is blocked by inhibitors of both PI3K and mTOR. l-Arg, but not d-Arg, activates MTOR cell signaling via phosphorylation of RPS6K and RPS6.

The effects of Arg on oTr cell proliferation are due in part to its metabolism to NO via NOS1/NOS2 and its metabolism by arginase to ornithine, which is converted by ODC1 to polyamines.^41^ Two NO donors, *S*-nitroso-*N*-acetyl-dl-penicillamine (SNAP) and diethylenetriamine NONOate (DETA), increased proliferation of oTr cells, as did putrescine, a polyamine. Both l-NAME (NOS inhibitor) and nor-NOHA (arginase inhibitor) decrease oTr cell proliferation by reducing NO and polyamines produced by oTr cells. Thus, both NO and polyamines stimulate proliferation and migration of oTr cells, but neither of the inhibitors of Arg metabolism fully suppress effects of Arg. This is likely because Arg may act via other cell signaling pathways, such as Rac activation,^42^ to stimulate cell proliferation and migration. Arg can also activate mitogen-activated protein kinase/extracellular-signal–regulated kinase (MEK/ERK) signaling, but the mechanism(s) whereby Arg acts on mammalian cells to activate mTORC1/mTORC2 and/or MEK/ERK is unknown.^43^

## Response of ovine conceptus explant cultures to select nutrients

Due to the possibility that oTr cells have an altered phenotype, effects of select nutrients were confirmed using day-16 sheep conceptus explant cultures. The abundance of transcripts for *mTOR*, *RPS6K*, *RPS6*, *EIF4EBP1, NOS1*, *NOS2*, *NOS3*, *GCH1, ODC,* and *IFNT* in conceptuses in control and in nutrient-supplemented medium was determined.^41^ Only conceptuses treated with Arg increased expression of *GCH1* mRNA. However, compared with control conceptus explant cultures, Arg increased total and phosphorylated forms of MTOR, RPS6K, RPS6, and EIF4EBP1, as well as IFNT, ODC1, NOS2, and NOS3 proteins. Leu increased total and phosphorylated mTOR, RPS6K, RPS6, and EIF4EBP1, but not IFNT, ODC1, NOS2, NOS3, or GCH1 proteins in conceptuses. Glucose increased total and phosphorylated forms of the MTOR cell signaling pathway proteins, as well as ODC1, NOS2, and GCH1 proteins. Gln increased total and phosphorylated RPS6, RPS6K, and EIF4EBP1 proteins, but only nonphosphorylated mTOR. Gln did not increase expression of ODC1, NOS2, or GCH1 proteins, but increased NOS3 protein. Thus, Arg-induced cell signaling via mTORC1 stimulates secretion of IFNT in a fast forward loop as IFNT increases expression of cationic amino acid transporters to deliver more Arg into the uterine lumen to enhance conceptus development and increased secretion of IFNT may increase expression of cationic amino acid transporters for transport of Arg.

## Exogenous P4 advances elongation of ovine conceptuses and transport of select nutrients into the uterine lumen

Growth and development of the conceptus is dependent on uterine LE/sGE and middle-to-deep GE to produce histotroph in response to P4 effects likely mediated via progestamedins and dependent on IFNT,^19^^,^^20^ as well as prostaglandins.^44^ A delay in the increase in circulating concentrations of P4 during metestrus and diestrus is associated with retarded conceptus development and reduced or delayed secretion of IFNT on day 17 in cattle.^45^^–^^48^ This adversely affects secretion of IFNT, which increases coordinately with elongation of the conceptus.^19^

Administration of exogenous P4 at 36 h after onset of estrus, that is, about 6 h postovulation, has been reported to advance conceptus development and IFNT secretion in sheep and cattle. Early P4 accelerates conceptus development and advances expression of uterine genes that favor survival, growth, and development of the conceptus.^49^^–^^53^ The early P4 treatment (1) advances the time of downregulation of PGR in uterine epithelia and onset of secretion, as well as abundance of IFNT; (2) increases abundance of secreted proteins such as galectin 15 (LGALS15), cathepsin L (CTSL), gastrin-releasing protein (GRP), stanniocalcin, and insulin-like growth factor binding protein 1 (IGFBP1) by uterine LE/sGE;^49^^,^^51^^,^^54^^–^^57^ (3) increases expression of *FGF10* and, to a lesser extent, *MET* mRNA, suggesting that FGF10 is the primary uterine stromal cell-derived progestamedin;^51^ (4) increases *MET* mRNA to increase responsiveness of uterine LE/sGE to HGF to enhance conceptus development as FGFR_2IIIb_ and MET are expressed by uterine epithelia and trophectoderm;^51^^,^^58^^,^^59^ (5) decreases tight-junction associated proteins in uterine LE that facilitates paracellular trafficking and/or transport of stromal and serum-derived molecules;^50^ (6) increases total recoverable glucose, aspartic acid, asparagine, serine, alanine, Gln, beta-alanine, citrulline, Arg, and lysine in the uterine lumen on day 9;^52^ (7) increases *SLC2A1* and *SLC5A1* mRNAs and proteins in uterine LE/sGE for glucose transport; and (8) increases *SLC7A2* mRNA in uterine LE/sGE for transport of cationic amino acids, particularly Arg.^52^

In cows, a 3-fold increase in circulating P4 increased recovery rates of blastocysts and a 2.3-fold increase in blastocyst size on day 13 of pregnancy, as well as increasing the frequency of elongated conceptuses on day 16 of pregnancy.^53^^,^^60^ These effects of P4 on conceptus development are mediated via the endometrium.^61^ Early P4 treatment in cattle advances downregulation of PGR^62^ and increases expression of genes for nutrient transport including *SLC5A1* (glucose transporter), nutrient availability such as *DGAT2* (diacylglycerol-*O*-acyltransferase for synthesis of triglycerides), *MSTN* (myostatin or growth/differentiation factor 8), which affects embryonic development and muscle mass, *FABP* (fatty acid–binding protein), and *CRYGS* (crystalline gamma-s for development of the lens in the eye).^63^ Forde *et al*. also found increased expression of *CTGF* (connective tissue growth factor), *LPL* (lipoprotein lipase), and *SLC5A1* (sodium-dependent glucose transporter) mRNAs in response to high concentrations of P4.^64^ Thus, P4 modifies the uterine environment by modifying the composition of histotroph to advance and enhance conceptus development in cattle.

## *In vivo* effects of Arg on successful outcomes of pregnancy

Recognition of the importance of Arg for survival and growth of the conceptus prompted studies to determine whether Arg increases successful pregnancy outcomes in animals and humans.^22^ Dietary supplementation with Arg-HCl increases fetal survival in gilts,^65^ and embryonic survival and litter size in rats.^66^ In ewe models of both undernutrition-induced and naturally occurring intrauterine growth retardation, intravenous administration of Arg-HCl enhanced fetal growth.^67^^,^^68^ Also, in women with intrauterine growth retardation of their fetus at week 33 of gestation, daily intravenous infusions of arginine increased birth weight at term.^69^

## MTOR cell signaling activated by secreted phosphoprotein 1 and integrins

Attachment and migration of trophectoderm cells are hallmarks of conceptus development and implantation in mammals. Secreted phosphoprotein 1 (SPP1) in the uterus binds integrins on conceptus trophectoderm and uterine LE to affect cell–cell and cell–matrix interactions. SPP1 induces motility in human trophoblast cells through mTOR signaling and rapamycin inhibits F-actin reorganization and phosphorylation of focal adhesion proteins stimulated by IGF1 such as focal adhesion kinase (FAK).^70^^,^^71^ These results were among the first to indicate SPP1-induced mTOR signaling in key events of pregnancy. Therefore, we identified relationships and crosstalk between multiple membrane and intracellular cell signaling cascades activated by SPP1, including mTOR, and integrin binding to oTr cells that control proliferation, migration, attachment, and adhesion in conceptuses during the peri-implantation period of pregnancy.^39^ SPP1 binds ITGAV:ITGB3 and possibly ITGA5:ITGB1integrin heterodimers to induce focal adhesion assembly, a prerequisite for adhesion and migration of oTr cells, through activation of (1) p70S6K via crosstalk between mTOR and MAPK pathways; (2) mTOR, PI3K, MAPK3/MAPK1 (ERK1/2), and MAPK14 (P38) signaling to stimulate oTr cell migration; and (3) focal adhesion assembly and myosin II motor activity to induce migration of oTr cells.^39^ These cell signaling pathways act in concert to mediate adhesion, migration, and cytoskeletal remodeling of trophectoderm cells essential for expansion and elongation of conceptuses and attachment to uterine LE for implantation.

## Key roles for fructose in development of ungulate conceptuses

**Figure 1:**
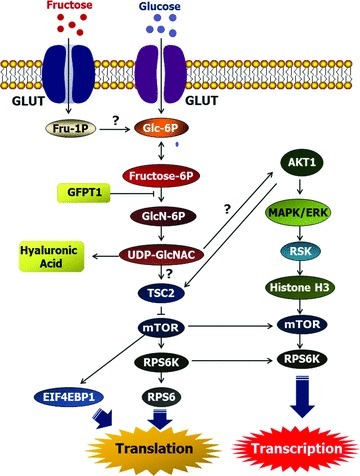
Schematic diagram of the glutamine:fructose-6-phosphate amidotransferase 1 (GFPT1)–mediated MTOR signaling pathway affected by glucose and fructose in porcine trophectoderm cells. Available evidence from our study indicates that fructose and glucose are metabolized via GFPT1 in the hexosamine biosysthesis pathway and activate mTOR-RPS6K and mTOR-EIF4EBP1 signal transduction cascades for porcine trophoblast cell proliferation and mRNA translation, as well as synthesis of glycosaminoglycans such as hyaluronic acid. Fru, fructose; Glc, glucose; GLUT, glucose/fructose transporter; Glc-6P, glucose-6-phosphate; Fru-1P, fructose-1-phosphate; Fru-6P; GlcN-6P, *N*-acetylglucosamine-6-phosphate; UDP-GlcNAC, UDP-N-acetylglucosamine; GFPT1, glutamine-fructose-6-phosphate transaminase 1; TSC2, tuberous sclerosis 2; mTOR, mechanistic target of rapamycin; RPS6K, ribosomal protein S6K; RPS6, ribosomal protein S6; EIF4EBP1, eukaryotic translation initiation factor 4E-binding protein 1; AKT1, protooncogenic protein kinase Akt; MAPK/ERK, mitogen-activated protein kinase/extracellular signal-regulated kinase.

In ungulate species (e.g., pigs and sheep) and cetaceans (e.g., whales), glucose is transported into the uterus and that which is not used for energy metabolism is converted to fructose by the placenta.^72^ However, the role of fructose is unclear although it is the most abundant hexose sugar in fetal blood and fluids of ungulate and cetacean species of mammals. Fructose has been studied in detail with respect to conceptus growth and development because it is not metabolized via the glycolytic pathway or the Krebs cycle. However, we recently reported that glucose and fructose are equivalent in being metabolized via the hexosamine pathway (Fig. 1) to stimulate mTOR cell signaling to increase cell proliferation and mRNA translation and to increase production of glycosaminoglycans critical for growth of the conceptus.^73^ Further, fructose may be metabolized via the pentose phosphate pathway to produce reducing equivalents, ribose sugars, and fatty acids for synthesis of lipids.^74^^–^^77^

## Summary and conclusions

This review focuses on the roles of P4 and IFNT in effecting gene expression in the uterine endometrium for secretory proteins and for transporters for delivery of nutrients into the uterine lumen to ensure conceptus development and successful outcomes of pregnancy. SPP1 and select nutrients, such as arginine, activate the mTOR nutrient-sensing pathway and focal adhesion assembly necessary for growth, development, and differentiation of the trophectoderm during the critical peri-implantation period of pregnancy. Early exogenous P4 accelerates conceptus development, increases abundance of select nutrients, induces early downregulation of PGR in uterine epithelia, and advances onset of secretion of IFNT for pregnancy recognition signaling, and progestamedins for regulation of function of uterine epithelia. These effects of early P4 enrich uterine histotroph to advance conceptus development and successful pregnancy outcomes in animals and humans. In addition, IFNT alters metabolic pathways in rats to mitigate against obesity and diabetes and these unpublished results provide the basis for future research to understand mechanisms whereby this novel interferon can be used therapeutically to prevent or ameliorate progression of inflammatory diseases.
